# The Impact of Boron Carbide Nanoparticle (B4C-NPs) Toxicity on *Caenorhabditis elegans* Models

**DOI:** 10.3390/toxics13060492

**Published:** 2025-06-12

**Authors:** Sen-Ting Huang, Erin P. Bulaon, Kai-Jie Yang, Adriana Taw, Lemmuel L. Tayo, Ping-Heng Hsieh, Jen-Hsiung Tsai, Jian-He Lu, Jheng-Jie Jiang, Hsing-Hsien Wu, How-Ran Chao

**Affiliations:** 1 Department of Environmental Science and Engineering, College of Engineering, National Pingtung University of Science and Technology, Neipu Township, Pingtung County 912, Taiwan; marssjason@gmail.com (S.-T.H.); a0928951291@gmail.com (K.-J.Y.); pinghenghsieh@mail.npust.edu.tw (P.-H.H.); tsaijh@mail.npust.edu.tw (J.-H.T.); 2 Department of Internal Medicine, Pingtung Veterans General Hospital, Pingtung City, Pingtung County 900, Taiwan; 3 School of Chemical, Biological, and Materials Engineering and Sciences, Mapúa University, Manila 1002, Philippines; epbulaon@gmail.com (E.P.B.); adrianabt1024@gmail.com (A.T.); or Lltayo@mapua.edu.ph (L.L.T.); 4 Department of Biology, School of Medicine and Health Sciences, Mapúa University, Makati 1200, Philippines; 5 Center for Agricultural, Forestry, Fishery, Livestock and Aquaculture Carbon Emission Inventory and Emerging Compounds, General Research Service Center, National Pingtung University of Science and Technology, Neipu Township, Pingtung County 912, Taiwan; toddherpuma@mail.npust.edu.tw; 6 Advanced Environmental Ultra Research Laboratory (ADVENTURE) & Department of Environmental Engineering, Chung Yuan Christian University, Taoyuan 320, Taiwan; jjjiang@cycu.edu.tw; 7 Department of Surgery, Tainan Municipal Hospital (Managed by Show Chwan Medical Care Corporation), Tainan 701, Taiwan; kenwu489@gmail.com; 8 Department of Nursing, Chung Hwa University of Medical Technology, Tainan 717, Taiwan; 9 School of Dentistry, College of Dental Medicine, Kaohsiung Medical University, Kaohsiung City 807, Taiwan

**Keywords:** nanotoxicity, boron carbide nanoparticles, *Caenorhabditis elegans*, antioxidants, neurotransmitter, genotoxicity

## Abstract

Boron carbide (B4C) is a widely recognized ceramic prized for its remarkable properties, including exceptional hardness, low density, and excellent chemical and mechanical stability. To date, limited research has explored the possible health risks associated with B4C nanoparticles (B4C-NPs). This study utilized a *Caenorhabditis elegans* (*C. elegans*) in vivo model to investigate the toxicological effects of B4C-NPs at concentrations of 40, 80, 160, and 320 mg/L. Larval nematodes were subjected to prolonged exposure, and their locomotion (head thrashing and body bending), reproduction (brood size), development (body length), lifespan, and gene expression (linked to oxidative stress, metal detoxification, apoptosis, and neurotransmitter synthesis) were assessed. Regarding survival rates, lethality was significantly increased to 5.41% at 320 mg/L of B4C-NPs and lifespan was significantly shortened across all concentrations compared with the controls. Development and reproduction showed slight reductions between 40 and 320 mg/L, while locomotion was markedly impaired at the doses from 80 to 320 mg/L. Gene expression related to antioxidants, apoptosis, cell cycle arrest, neurotransmitter synthesis, and metal detoxification rose significantly at 160–320 mg/L in *C. elegans*, suggesting that B4C-NPs may induce reproductive and neurological toxicity, delay development, reduce lifespan, and potentially cause genotoxicity in *C. elegans*.

## 1. Introduction

Boron carbide (B4C) is considered one of the hardest synthetic materials, widely applied in abrasive and wear-resistant products due to its exceptional properties [1]. With its high hardness, low density, great chemical resistance, and excellent nuclear properties, B4C nanoparticle (NP) have been utilized in the nuclear, microelectronics, and biomedical industries. These attributes make it a top choice for applications such as protective armors, cutting tools, grinding equipment, and nuclear technology. As biomedical components, synthesized B4C-NPs exhibit high-quality structural, optical, and electrical properties, making them promising candidates for developing photodetectors [2]. Moreover, recent studies have indicated their potential in cancer therapy, such as boron neutron capture therapy (BNCT), with opportunities for further enhancement to improve therapeutic delivery abilities [3,4].

NPs, owing to their small size, large surface area, and inhomogeneous electron distribution or uneven spatial arrangement of electrons across their surfaces, have found broad applications in consumer products, medicine, and soil and aquatic environments, which have included data storage, drug delivery, and environmental purification [5,6]. However, these same properties enable NPs to penetrate physiological barriers in organisms, potentially causing harmful effects such as cell death, oxidative stress, DNA damage, apoptosis, epigenetic regulation, and induced inflammatory responses [5,6]. Among these nanomaterials, B4C-NPs have attracted attention for their unique properties and potential biomedical applications, yet their safety remains a critical concern.

A cytotoxicity evaluation on human pulmonary alveolar epithelial cells (HPAEpiCs) revealed that of B4C-NPs exhibited low toxicity at moderate concentrations, indicating their potential safety for pharmacological and medical applications [7]. Additional studies on other cell lines, such as the human epithelial malignant melanoma cell line A-375 and mouse embryo fibroblast cell line NIH3T3, indicated that B4C-NPs displayed less cytotoxicity compared with other NPs, suggesting minimal adverse effects on cells [8]. Moreover, B4C-NPs influence the amino acid biosynthesis process, interfere with the visualization of protein–RNA complexes, and modulate signal protein expression, potentially exerting a negative impact on tumor growth, progression, and drug resistance [7,8]. Although these findings suggest that B4C-NPs can be used as a safe nanomaterial in both pharmacological and medical applications, their safety profile may vary with exposure conditions, necessitating further investigation into their effects on human health.

*Caenorhabditis elegans* (*C. elegans*) is a small, free-living transparent nematode that inhabits temperate soil environments. It is widely used as a model organism in scientific research due to its simple anatomy, ease of cultivation, short lifespan, and fully sequenced genome. For instance, it has been utilized as an experimental model for toxicological research, such as studies on nanoparticles and fine particulate matter toxicity [9,10,11,12,13,14]. According to previous studies, NPs induce various effects in *C. elegans*, such as development changes, intestinal function, immune response, and neuronal function [15].

We hypothesized that B4C-NPs would induce toxicological effects in *C. elegans*, affecting locomotion, reproduction, development, survival rates, lifespan, and gene expression related to oxidative stress, apoptosis, DNA damage, neurotransmitter synthesis, and metal detoxification. Larval *C. elegans* were exposed to 40, 80, 160, or 320 mg/L B4C-NPs, and a control group was also established. The current study analyzed locomotion behavior through the frequence of head thrashing and body bending, reproduction through brood size, and development through body length analysis. Survival rate was assessed via lethality, lifespan via longevity, and gene expression via quantitative real time-PCR. Our study found that B4C-NPs can induce reproductive toxicity and decrease motor function and survival rate. These findings may imply that B4C-NPs can cause detrimental effects on *C. elegans*, and further studies of other physiological parameters must be conducted in the future.

## 2. Materials and Methods

### 2.1. Preparation of Boron Carbide Nanoparticles

The B4C-NPs were originally purchased from Sigma Aldrich (product number: 378119, powder, <10 μm, 98%). They were modified and remanufactured by a national military research institute in Taiwan according to the protocol described in a previous report, with minor modification [16], before being gifted for use in this study. After mixing 0.64 g of B4C-NPs and 1 L of distilled water, the mixture was placed in an electromagnetic stirrer along with a magnet stir bar and stirred for 24 h. The B4C-NP suspensions were kept by storage at 4 °C in the dark. Prior to use, the B4C-NPs were then dispersed in K medium (30 mM g KCl (Avantor Performance Materials, Ltd., Gyeonggi-do, South Korea), 50 mM NaCl, and 3 mM CaCl_2_ in 1 L ddH_2_O). The primary particle size of the B4C-NPs ranged from 0.4 to 0.8 μm and the dispersion particle size was approximately within the same range after the dispersion procedure. No particle dissolution or leaching of impurities in the K medium was observed. The nanoparticle characterization of B_4_C-NPs referenced the Manimaran report [16], which is briefly described in the Supplementary Materials. Afterwards, the mixture was transferred into a 15 mL centrifuge tube and placed in an oscillator before use.

### 2.2. Reagents and Raw Materials

Nematode growth medium (NGM) plates were produced using 2.4 g of NaCl, 24 g of pectin, and 2 g of digestive protein, along with 780 mL of ultrapure water, which were mixed in a 1000 mL serum vial and sterilized for 50 min. After sterilization, the mixture was transferred to a circulating water bath and cooled to 55 °C. Finally, 0.8 g of CaCl_2_, MgSO_4_ and cholesterol, and 20 mL of phosphate were added to the mixture, which was then exposed to ultraviolet (UV) light for 24 h before the Petri dishes were placed in a refrigerator. *Escherichia coli* (*E. coli*) OP50, which was utilized as food for the *C. elegans*, was prepared through the following procedure. First, the *E. coli* was placed in the centrifuge for 10 s to form a bacterial pellet. Subsequently, 10 µL of the resuspended pellet was transferred to 5 mL of Luria– Bertani (LB) broth and incubated at 37 °C with shaking at 200 rpm for 16–18 h to promote bacterial growth. The culture was then exposed to ultraviolet (UV) light for 24 h to inactivate the bacteria, ensuring safety for nematode feeding, and stored at −20 °C until use.

### 2.3. Chunking and Bleaching of C. elegans

Sterilized tweezers were used to move a chunk of agar from an old plate to a fresh plate seeded with *E. coli* OP50, facilitating *C. elegans* growth and collection for further experiments. After 48 h of incubation, the culture media with the greatest numbers of eggs and adult nematodes were selected for bleaching. The culture medium was rinsed with sterilized water, and the water containing the eggs was then transferred into 15 mL centrifuge tubes; this was repeated until the tube was filled. Afterwards, the centrifuge tubes were placed in the centrifuge at a speed of 2800 rpm for 2 min. The 14 mL upper layer of water was removed using a vacuum aspirator and filled with water again until 15 mL before placing the tube in the centrifuge to remove the *E. coli* residues. Then, using the vacuum aspirator, the upper layer was removed, leaving 3.5 mL of water containing the eggs and adult nematodes. Subsequently, 0.5 mL of 5 N NaOH and 1 mL household bleach (5% solution of sodium hypochlorite) were added, and the tube was shaken for 5 min. Afterwards, the tube was filled with water for 15 mL and then placed in the centrifuge. The upper layer of water was removed and replaced with sterilized water before placing the tube back in the centrifuge. Then, the excess water was removed, leaving the eggs in the lowest part of the tube. Finally, 4 mL of M9 buffer solution (7.56 g Na_2_HPO_4_ (Honeywell Fluka™, NJ, USA), 1.5 g KH_2_PO_4_ (Avantor Performance Materials, LLC, Radnor, PA, USA), 2.5 g NaCl, 0.5 mL 1 M MgSO_4_ (Avantor Performance Materials, Ltd., Gyeonggi-do, South Korea), 500 mL H_2_O) was added to the eggs and mixed; then, using a pipette, the mixture was transferred in a Petri dish before it was placed in a freezer for 24 to 48 h.

### 2.4. Nanoparticle Exposure

After the bleaching process, 600 µL of *C. elegans* eggs were transferred using a pipette into a 2 mL tube and were left to rest for 30 min to allow egg sedimentation and facilitate removal of the bleaching solution. Next, the supernatant was carefully removed, and the eggs were washed 2–3 times with sterile M9 buffer. Then, the cleaned eggs were transferred to NGM agar plates seeded with *E. coli* OP50 and incubated at 22 °C. After the synchronized L1 nematodes developed into L3 or young L4 nematodes, they were rinsed from the plate with K medium and centrifuged at 2500 g for 4 min. The worm pellet was resuspended in K medium, and approximately 200 L3/young L4 nematodes were dispensed into each well of a 12-well plate. After 24 h of starvation, the nematodes were rinsed from the plate with 600 µL of K medium, then pipetted into a 2 mL tube and allowed to settle for 30 min. After removing 550 µL of the supernatant, 40 µL of K medium containing *C. elegans* was pipetted into a new tube. Then, 10 µL of the untreated control and 10 µL of B4C-NPs were added, adjusting the final concentrations to 40, 80, 160, and 320 mg/L, respectively. After mixing the B4C-NPs and *C. elegans* thoroughly and allowing them to settle for 30 min, the mixture was evenly distributed onto agar plates seeded with *E. coli* for B4C-NPs exposure analysis.

### 2.5. Growth, Reproductive, Lifespan, and Lethality Assays of C. elegans

The worms were exposed to B4C-NPs at concentrations of 0–320 mg/L for at least 48 h from L1-larvae to young adults’ stages, while incubated at 20 °C. For the growth measurement assay, *C. elegans* were exposed to the test conditions for 72 h until late L4 stage. Subsequently, the L4 nematodes were mounted into a glass pad containing 10% formalin solution. Body length analysis (head to tail) was captured using an Olympus SZX10 dissecting microscope, and body lengths were measured using imaging software. Three independent experiments were performed and, for each experiment, at least 20 control and treated worms were analyzed (*n* = 60 worms per concentration).

For the reproductive assay, *C. elegans* were exposed to B4C-NPs for 48 h. Subsequently, one L3/young L4 nematode was transferred to a new NGM agar plate seeded with *E. coli* OP50 (10 nematodes per concentration, repeated three times, *n* = 30 per concentration) and incubated at 20 °C for 48 h before removal. The eggs on these plates were allowed to hatch, and the resulting larvae were incubated at 20 °C until reaching the L4 stage for accurate offspring counting. Finally, the number of progenies per nematode was counted.

For the lethality assay, *C. elegans* were subjected to acute (24 h) exposure to different concentrations of B4C-NPs and then transferred to fresh NGM plates seeded with *E. coli* OP50 (10 nematodes per concentration, repeated three times, *n* = 30 per concentration) and incubated at 20 °C. At the end of the exposure period, worms were enumerated and assessed as alive or dead under a microscope; they were deemed dead if they showed no response to touch with a small, metal wire. For the lifespan assay, synchronized young adult worms were placed on NGM agar plates seeded with *E. coli* OP50 and treated with different concentrations of B4C-NPs. *C. elegans* were transferred every other day to fresh NGM plates, and their survival was scored by gentle prodding with a platinum wire to test for live or dead worms for a duration of 24 days. Worms that stopped pharyngeal pumping and did not move, even after repeated prodding, were classified as dead and removed from the plates. Those that had crawled onto the plate edges and died away from the agar were excluded from the analysis. Both the lifespan and lethality assays were conducted in triplicate for each concentration.

### 2.6. Locomotion Assay

The locomotive behavior of the nematodes was evaluated using head thrashing and body bending assays. A successful head thrash was defined as a change in the direction of bending at the mid-body bend, while body bending was described as a change in the direction of the nematodes corresponding to the posterior bulb of the pharynx along the y axis, assuming that nematode was traveling along the x axis. For head thrashing assessment, the nematodes were individually selected and transferred to a Petri dish with a new culture medium, M9. Then, the movements of the nematodes were recorded using a dissecting microscope for 1 min; 30 worms per concentration were evaluated. For the body bending assay, the nematodes were individually placed on a new NGM plate. Then, water was added to the plate before recording the movement under a dissecting microscope for 20 s. The frequency of body bending was counted for the exposed and non-exposed worms; 30 worms per concentration were evaluated.

### 2.7. Gene Expression Assay

Total RNA was extracted from B4C-NP-exposed nematodes to analyze mRNA levels. The nematodes were washed down before adding 1 mL of TRIzol reagent to lyse the nematodes and stabilize RNA by inhibiting RNases. Then, the isolated RNA was placed in a −80 °C freezer to preserve RNA integrity, and it was placed in a container full of ice to prevent degradation when removed from the freezer. The RNA was extracted from the cDNA by adding 70 µL of 1-bromo-3-chloropropane at room temperature to separate the aqueous phase containing RNA from organic components. Then, the mixture was placed in a centrifuge at 13,000 rpm for 12 min at 4 °C to enhance phase separation. The upper layer of the mixture was transferred to a sterilized 1.7 mL microcentrifuge tube, and an equal amount of isopropanol was then added to precipitate RNA, with incubation at −80 °C for 10 min to maximize yield. Afterwards, the RNA pellet was obtained by centrifugation at 13,000 rpm for 12 min at 4 °C, washed with 750 µL of 75% of ethanol to remove impurities, and centrifuged again under the same conditions. Then, the supernatant was removed, and the tubes were air-dried at room temperature until the droplets of water dried. After drying, an appropriate amount of diethyl pyrocarbonate was added to dissolve the RNA; then, the RNA was quantified using spectrophotometric setup value of 260/280, and the recording was inverted. Next, 1 μg RNA was converted into cDNA using the High-Capacity cDNA Reverse Transcription kit (Gibco, Life Technologies, Carlsbad, CA, USA). For real-time PCR, 50 ng of cDNA, 5 µL of SYBR Green, 3 µL of nuclease-free water, and 1 µL each of forward (5mM) and reverse (5 mM) primer were all mixed in the 96-well plates and sealed with 96-well strips. Then, the mixture was placed in an Applied Biosystems PRISM 7500 Real-Time PCR System. Afterwards, the cycle threshold (Ct) was analyzed and the 2^−ΔΔCT^ equation was used to determine the mRNA difference between the control and experimental groups.

In this study, several genes were selected to examine the impact of B4C-NPs on adverse effects in *C. elegans*. The expression of genes was divided into 4 groups including antioxidants, apoptosis or cell cycle arrest, neurotransmitter synthesis or serotonin synthesis, and metal detoxification. The first set of genes, including oxidative stress [superoxide dismutase-1 (sod-1), sod-2, sod-3, sod-4, sod-5, ctl-1, ctl-2, ctl-3, and skinhead-1 (skn-1), were utilized to determine oxidative stress in the *C. elegans* [17,18,19,20]. Oxidative stress is the imbalance between the production of reactive oxygen species (ROS) and antioxidant defenses, which results in tissue injury [21]. The second set of genes including cell death abnormality 3 (ced-3), ced-4, ced-9, ced-13, human HUS1 related 1 (hus-1), and C. Elegans P-53-like protein 1 (cep-1) genes were those involved in DNA damage-induced cell cycle arrest, cell death, or apoptosis [22,23,24,25]. To preserve genomic stability in response to DNA damage, multicellular organisms initiate checkpoints that trigger either cell cycle arrest or apoptosis. Apoptosis occurs when cells are nonfunctional, activating an intracellular death program, also known as programmed cell death [26]. The third set of genes [cationic amino acid transporter-1 (cat-1), cat-4, modulation of locomotion defective 5 (mod-5), and tryptophan hydroxylase 1 (tph-1)] play a key role in the regulation of neurotransmitter synthesis, including the transportation and biosynthesis of serotonin [27,28,29,30,31,32]. Serotonin is responsible for regulating human behavioral processes including digestion, wound healing, sleep, etc. [33]. The fourth set of genes including metallothionein 1 (mtl-1) and mtl-2 contribute to the metal detoxification in *C. elegans*, and cytochrome P450 (cyp) 35a2 gene is responsible for various biological processes such as xenobiotic metabolism and stress response [34,35]. We also quantified the expression of actin mRNA as an internal control. Sequences for the primers used are shown in Table 1.

### 2.8. Statistical Analysis

The data obtained in the entire study were compiled using Microsoft Excel and statistically analyzed using GraphPad Prism 5. The values were expressed as means ± standard error mean (Mean ± SEM), and t-tests were used to determine the significance of distinct groups, with *p* < 0.05 and 0.01 representing statistical significance. Lastly, the graphs and plots were created using GraphPad Prism 5.

## 3. Results

### 3.1. Toxicological Effects of B4C Nanoparticles on Growth, Lethality, and Lifespan in C. elegans

**Figure 1:**
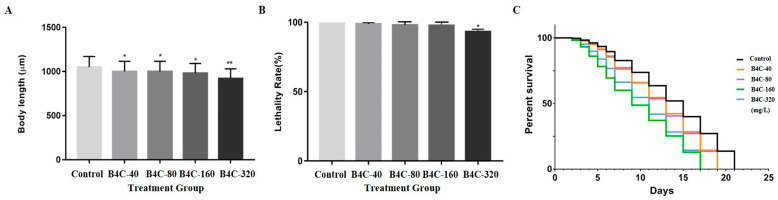
Tests of development, survival rates, lifespan of *C. elegans* after prolonged exposure to B4C-NPs at concentrations of 40 (B4C-40), 80 (B4C-80), 160 (B4C-160), and 320 (B4C-320) mg/L as well as the untreated control: (**A**) body length (development), (**B**) lethality (survival rate), and (**C**) longevity (lifespan). Bars show mean ± SD. Significant differences expressed as * *p* < 0.05 and ** *p* < 0.01.

Figure 1A shows the growth rate of *C. elegans* exposed to B4C-NPs, compared with the control group. The data demonstrate that exposure to concentrations of 40 and 320 mg/L B4C-NPs significantly retarded the growth of *C. elegans* compared with the control group. Figure 1B represents the lethality rate of the *C. elegans* after being exposed to B4C-NPs. The results revealed a decrease of 5.41% for exposed nematodes at 320 mg/L, suggesting a dose-dependent reduction in viability as the nanoparticle concentration increased. Lifespan, as a toxicological endpoint, was assessed over 24 days (Figure 1C). The lifespan of *C. elegans* was significantly reduced by approximately 74%, beginning at 9 days after treatment with 160 and 320 mg/L B4C-NPs, compared with the control group (Figure 1C). These results indicate that exposure to 160 or 320 mg/L B4C-NPs may have had some impact on the worms’ growth and lifespan.

### 3.2. B4C-NPs Impair Locomotion and Progeny Production in C. elegans

**Figure 2:**
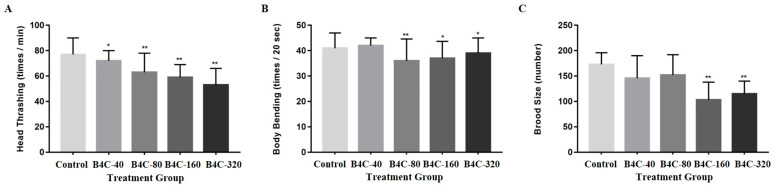
Tests of locomotion and reproduction of *C. elegans* after nematodes’ prolonged exposure to B4C-NPs at concentrations of 40 (B4C-40), 80 (B4C-80), 160 (B4C-160), and 320 (B4C-320) mg/L as well as the untreated control. (**A**) head thrashing, (**B**) body bending, and (**C**) reproduction. Bars show mean ± SD. Significant differences expressed as * *p* < 0.05, and ** *p* < 0.01.

The effects of B4C-NPs on the locomotive and reproductive performance of *C. elegans* are shown in Figure 2. Figure 2A presents the changes in head thrashing behavior of *C. elegans* at various concentrations of B4C-NPs. The results indicate significant reductions in head thrashing frequency of 9.18%, 16.87%, 24.07%, and 29.28% at concentrations of 40, 80, 160, and 320 mg/L, respectively, compared with the untreated control. Regarding body bending, as shown in Figure 2B, the findings revealed declines in body bending frequency of 14.18%, 12.53%, 8.98% at 80, 160, and 320 mg/L, respectively. These findings suggest that B4C-NPs negatively impacted the locomotion of *C. elegans*, particularly at higher concentrations from 80 to 320 mg/L. Figure 2C focuses on the impact of B4C-NPs on the *C. elegans*’ production of progeny. According to the observed brood sizes, there were significant changes in the reproductive performance of the nematodes exposed to concentrations from 160 to 320 mg/L. The findings show that reproduction declined by 40.52% and 33.53% at 160 and 320 mg/L, respectively, compared with the control group.

### 3.3. Impact of B4C-NPs on Oxidative Stress Genes in C. elegans

**Figure 3:**
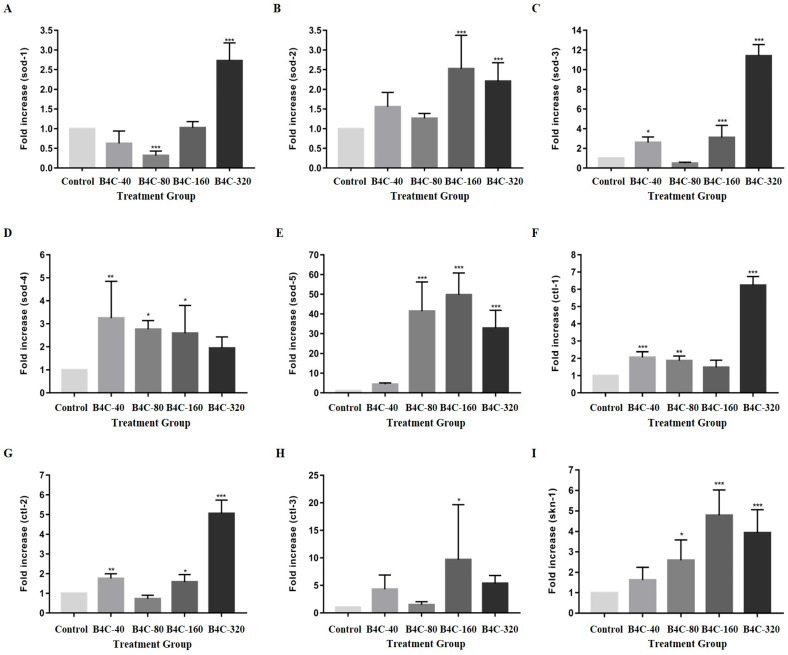
Gene expression of (**A**) sod-1, (**B**) sod-2, (**C**) sod-3, (**D**) sod-4, (**E**) sod-5, (**F**) ctl-1, (**G**) ctl-2, (**H**) ctl-3, (**I**) skn-1 in *C. elegans* after nematodes were exposed to B4C-NPs (40 (B4C-40), 80 (B4C-80), 160 (B4C-160), and 320 (B4C-320) mg/L and control). Bars show mean ± SD. Significant differences expressed as * *p* < 0.05, ** *p* < 0.01, and *** *p* < 0.001.

The results from the gene expression assay are shown in Figure 3. In the nematodes exposed to B4C-NPs at 160 mg/L, gene expression increased by 153.63% (sod-2), 208.33% (sod-3), 166.37% (sod-4), 6300.05% (sod-5), 57.73% (ctl-2), 1017.39% (ctl-3), and 386.74% (skn-1) compared with the control group. Additionally, increases of 179.9% (sod-1), 126.47% (sod-2), 1125.0% (sod-3), 99.9% (sod-4), 4125.04% (sod-5), 540.7% (ctl-1), 406.19% (ctl-2), 521.74% (ctl-3), and 297.96% (skn-1) were observed at 320 mg/L. These results indicate concentration-dependent and gene-specific responses to B4C-NPs in *C. elegans*. However, at 80 mg/L, reductions of 69.07% (sod-1) and 52.27% (sod-3) were noted. These findings indicate activation of robust antioxidant defense at higher B4C-NPs concentrations. Conversely, at 80 mg/L, reductions of 69.07% (sod-1) and 52.27% (sod-3) were noted. These results highlight the complex, dose-dependent interplay between B4C-NP exposure and oxidative stress regulation in *C. elegans*, with potential implications for NP toxicity and cellular defense mechanisms.

### 3.4. B4C-NPs Induce Apoptosis and DNA Damage Responses in C. elegans

**Figure 4:**
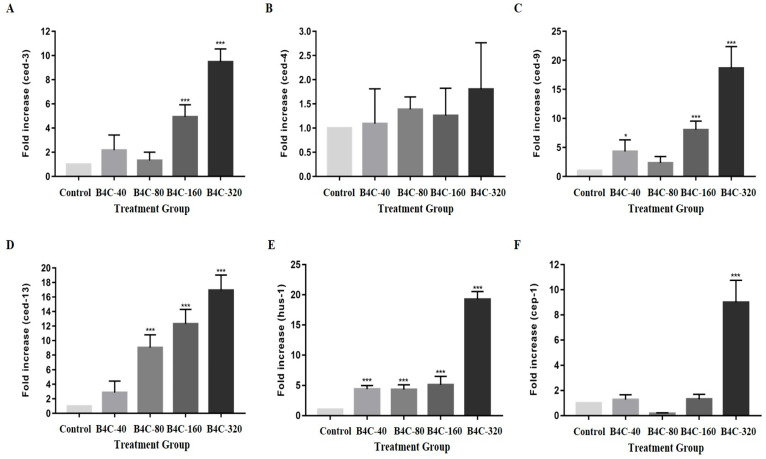
Gene expression of (**A**) ced-3, (**B**) ced-4, (**C**) ced-9, (**D**) ced-13, (**E**) hus-1, and (**F**) cep-1 in *C. elegans* after nematodes were exposed to B4C-NPs (40 (B4C-40), 80 (B4C-80), 160 (B4C-160), and 320 (B4C-320) mg/L and control). Bars show mean ± SD. Significant differences expressed as * *p* < 0.05 and *** *p* < 0.001.

As shown in Figure 4, the expression levels of apoptosis-related genes (ced-3, ced-4, ced-9, ced-13, hus-1, and cep-1) in *C. elegans* were significantly altered following exposure to various concentrations of B4C-NPs. At a concentration of 80 mg/L, there was a notable 88.00% decrease in the expression of the cep-1 gene compared with the control group. In contrast, exposure to 160 mg/L of B4C-NPs resulted in substantial increases in the expression of these genes, including ced-3 (432.69%), ced-9 (744.00%), ced-13 (1165.62%), and hus-1 (426.92%), compared with the untreated controls. Compared with the control groups, these increases were more pronounced at 320 mg/L, including in ced-3 (938.46%), ced-4 (82.80%), ced-9 (1832.00%), ced-13 (1640.62%), hus-1 (1888.47%), and cep-1 (848.00%). Additionally, positive changes in gene expression were also observed at lower concentrations. At 40 mg/L, increases in ced-3 (132.69%), ced-9 (344.00%), ced-13 (193.75%), and hus-1 (353.85%) were recorded, compared with the controls.

### 3.5. Effects of B4C-NPs on Neurotransmitter Synthesis-Related Gene Expression in C. elegans

**Figure 5:**
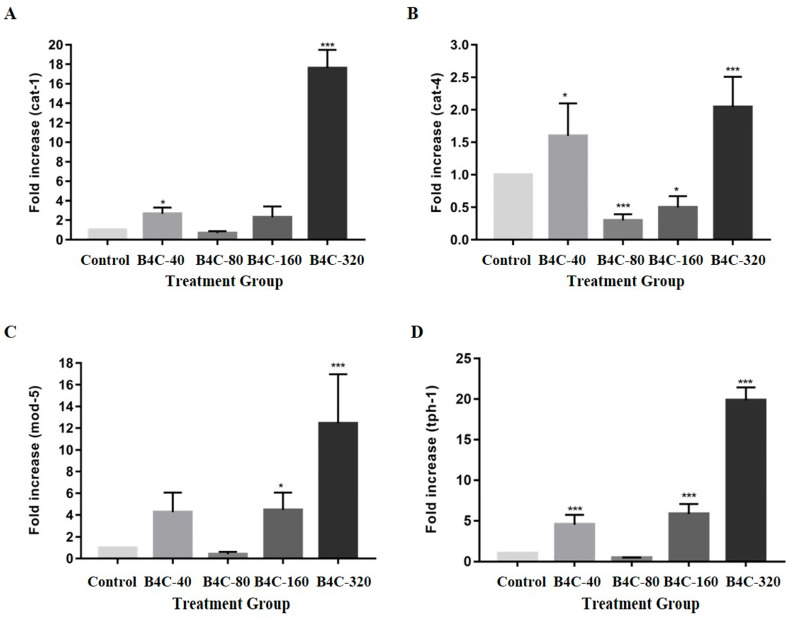
Gene expression of (**A**) cat-1, (**B**) cat-4, (**C**) mod-5, and (**D**) tph-1 in *C. elegans* when worms were exposed to B4C-NPs (40 (B4C-40), 80 (B4C-80), 160 (B4C-160), and 320 (B4C-320) mg/L and control). Bars show mean ± SD. Significant differences expressed as * *p* < 0.05 and *** *p* < 0.001.

As can be observed in Figure 5, exposure of *C. elegans* to 320 mg/L of B4C-NPs resulted in a significant increase in serotonin-related gene expression. Specifically, the expression levels of cat-1, cat-4, mod-5, and tph-1 increased by 1674.28%, 105.91%, 1105.13%, and 1960.00%, respectively, compared with the control group. Conversely, at 80 mg/L, the expression of these genes was significantly reduced, particularly for the cat-4 gene, which decreased by 70.45%. Other reductions at this concentration included those in mod-5 (56.41%) and tph-1 (56.00%), compared with the controls.

### 3.6. Influence of B4C-NPs on Metal Detoxification Genes in C. elegans

**Figure 6:**
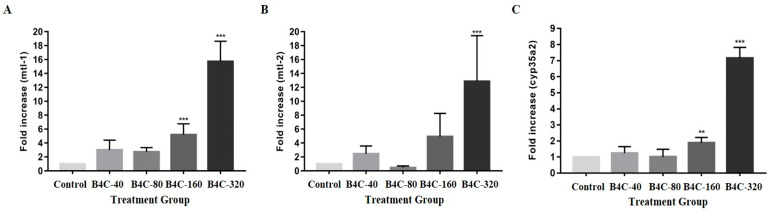
Gene expression of (**A**) mtl-1, (**B**) mtl-2, and (**C**) cyp35a2 in *C. elegans* after *C. elegans* following exposure to B4C-NPs (40 (B4C-40), 80 (B4C-80), 160 (B4C-160), and 320 (B4C-320) mg/L and control). Bars show mean ± SD. Significant differences expressed as ** *p* < 0.01 and *** *p* < 0.001.

As shown in Figure 6, the expression levels of the mtl-1, mtl-2, and cyp35a2 genes in *C. elegans* were significantly influenced by exposure to B4C-NPs. At a concentration of 320 mg/L, there were significant increases of 1625.80%, 1352.18%, and 636.36% in mtl-1, mtl-2, and cyp35a2 genes, respectively, compared with the control group. However, a negative change of 56.52% at 80 mg/L in mtl-2 was also observed.

## 4. Discussion

Few studies have used *C. elegans* as a model system to test the toxicity of B4C-NPs, and the same may be true for studies using rodent-based models. Using several toxicological endpoint assays in *C. elegans*—such as body length, lethality, lifespan, locomotive behavior, reproduction, and gene expression—we showed that nematodes with exposure to 320 µg/L of B4C-NPs had significantly reduced body length, lifespan, brood size, and frequency of head thrashing and body bending. Furthermore, this exposed dose (320 µg/L B4C-NPs) also upregulated mRNA expression of genes involved in antioxidant activity, apoptosis, neurotransmitter synthesis, and heavy metal detoxification.

Our study indicated that B4C-NPs (inorganic NPs) significantly increased survival rates and reduced lifespan in *C. elegans*, particularly at higher concentrations (Figure 1B,C), in contrast to in vitro cell line studies that have been employed to explore the cytotoxicity and molecular mechanisms of B4C-NPs at a cellular level [3,7,8]. Delong et al. (2016) [8] reported that the cytotoxicity of B4C was lower than that of ZnO NPs at concentrations of 25–50 mg/L in human A-375 and mouse NIH3T3 cell lines. Exposure to 50 mg/L of B4C-NPs also had no significant effect on the morphology of HeLa cells [8]. Additionally, B4C-NPs also reduced hemolysis in red blood cells [8]. Türkez et al. (2019) [7] found that significant toxicity of B4C-NPs toxicity in HPAEpiC cells line occured at 80 mg/L, with an IC_20_ value of 202.525 mg/L. Exposure to 320 mg/L of B4C-NPs resulted in nearly complete cell death across the culture population. At the IC_20_ concentration (202.525 mg/L), B4C-NPs primarily affected amino acid biosynthesis and the expression of developmental proteins. Similarly, Kozień et al. (2023) [3] analyzed the toxic effects of B4C-NPs on human and mouse cell lines, including phagocytic, normal, and tumor cell lines. They found that the IC_50_ value for B4C-NPs ranged between 50 and 100 mg/L in mouse macrophage cells, though non-malignant and tumor cell lines were less sensitive. Tumor cells were less sensitive to B4C-NPs than murine macrophages but more sensitive than human monocyte-like THP-1 cells [3]. Regarding NPs in *C. elegans* models, Tsai et al. (2021) [14] indicated that prolonged exposure of nematodes to low-dose graphene oxide (GO) NPs (carbon-based NPs) did not result in significant lethal effects. Another study also revealed that polypyrrole (Ppy) NPs (organic/polymeric NPs) had no adverse toxic effect on the survival rate of the exposed worms at any concentration (20, 100, and 500 mg/L) [36]. Similarly, an in vivo study reported that TiO_2_ NPs (inorganic NPs) had no significant effects on the survival rate of nematodes until 500 mg/L [37]. Our previous studies demonstrated that exposure to TiO_2_ NPs (0.0, 0.01, 0.1, 1.0, and 10 mg/L) had no effect on the survival rates of nematodes, regardless of the concentration [38]. However, our previous studies showed that exposure to ≥1 μg/L carboxylated single-walled carbon nanotubes (SWCNTs-COOH) (carbon-based NPs) could induce toxicity in nematodes that affected lifespan [11]. These findings suggest that B4C-NPs may have a higher toxicity profile compared with other Nps like GO, Ppy, and TiO_2_.

The current study showed that B4C-NPs significantly impaired body length in *C. elegans* (Figure 1A), indicating developmental toxicity. In contrast, the nematodes attained an average body length of approximately 1100 µm after Ppy NPs exposure, indicating that the Ppy NPs had no influence on the worms [36]. Similarly, our previous studies demonstrated that exposure to TiO_2_ NPs (0.0, 0.01, 0.1, 1.0, and 10 mg/L) had no effect on the body length of nematodes, regardless of the concentration [38]. However, our previous studies showed that exposure to ≥1 μg/L SWCNTs-COOH could induce toxicity in nematodes, affecting growth [11]. In conclusion, while B4C-NPs and carboxylated SWCNTs-COOH induce developmental toxicity in *C. elegans* by impairing body length, Ppy NPs and TiO_2_ NPs exhibit no adverse effects on growth.

Reproduction is a crucial endpoint in *C. elegans* because changes in progeny production can identify potential toxic effects on the organism. The reproductive performance of the nematodes significantly declined, as shown in Figure 2C, particularly at high doses of B4C-NPs. This finding is similar to the study by Mashock et al. (2016), in which they stated that production of progeny was reduced by high concentrations of copper oxide (CuO) NPs [39]. Another study indicated that prolonged exposure to GO NPs reduced progeny production in nematodes particularly for high doses of 10 mg/L [14]. Our previous studies demonstrated that 24 h exposure to 10 mg/L TiO_2_ or 0.1–1000 μg/L SWCNTs-COOH also induced reproductive toxicity in nematodes [11,38]. In contrast, exposure to the highest measured concentration of 500 μg/mL Ppy NPs did not cause reproductive toxicity in nematodes, indicating that Ppy NPs exhibit biocompatibility in *C. elegans* [36]. NPs may also cause *E. coli* aggregation due to their charge, further affecting the growth and reproduction of *C. elegans*, as described in a previous report [40]. In conclusion, high doses of certain Nps, such as B4C, CuO, GO, TiO_2_, and SWCNTs-COOH, significantly impair reproductive performance in *C. elegans*, while Ppy NPs demonstrate biocompatibility, highlighting the importance of material-specific toxicity assessments for evaluating nanoparticles’ safety.

As illustrated in Figure 2, B4C-NPs significantly delayed neurobehaviorial development such as head thrashing and body bending in *C. elegans* models in the present study. Effective and safe drug therapies for central nervous system (CNS) disorders remain a significant global challenge with regard to human health. Nanotechnology offers promising solutions for diagnosis and treating various CNS disorders by facilitating drug delivery across the blood–brain barrier (BBB) and enabling targeted delivery of multiple therapeutic agents to specific cell types [41,42]. However, research has shown that when NPs interact with cerebral blood vessel walls, they can stimulate endothelial cells to secrete pro-inflammatory cytokines, leading to a pronounced immune response [43]. These findings underscore the importance of carefully assessing the immunogenicity and potential neurotoxicity of NPs. Locomotion assays are among the most efficient methods for evaluating neurotoxicity in nematodes [9]. Tsai et al. (2021) reported that GO NPs caused an apparent decline in body bending and head thrashing [14]. Our previous studies revealed that 24 h exposure to 0.1–10 mg/L TiO_2_ or 0.1–1000 μg/L SWCNTs-COOH caused a concentration-dependent decline in the frequency of body bending and head thrashing in nematodes [11,38]. The findings of the current study also showed that the frequency of head thrashing and body bending in B4C-NPs-exposed nematodes significantly decreased compared with the control group (Figure 2A,B). Collectively, these results highlight the dual nature of NPs as both innovative tools for CNS therapy and potential sources of neurotoxic risk, necessitating thorough safety evaluations before clinical application.

In *C. elegans*, the genes sod-1, sod-2, sod-3, sod-4, sod-5, ctl-1, ctl-2, ctl-3, and skn-1 were used to assess oxidative stress [17,18,19,20]. As illustrated in Figure 3, in the present study, gene expression was used to determine oxidative stress (sod-1, sod-2, sod-3, sod-4, sod-5, ctl-1, ctl-2, ctl-3, and skn-1 genes); there was overexpression of all these genes, particularly at high concentrations of 320 mg/L. However, the extent of upregulation varied across treatment concentrations, probably due to differences in B4C-NP bioavailability, aggregation, or cellular uptake, which may influence gene regulation pathways differently. These results suggest that 80 mg/L may trigger compensatory mechanisms that suppress antioxidant genes (sod-1 and sod-3), whereas higher concentrations (160 and 320 mg/L) overwhelm these mechanisms, leading to robust upregulation. Excessive ROS and oxidative stress can cause oxidative damage and dysfunction in biomolecules such as DNA, proteins, and lipids, leading to cellular and tissue damage. Eliminating ROS with antioxidants may serve as an effective strategy for preventing disease progression. Our previous study demonstrated that exposure to 1000 μg/L SWCNTs-COOH significantly induced ROS generation in *C. elegans* and increased the mRNA expression of sod-1 and sod-3 [11]. Exposure to TiO2_2_ NPs significantly induced intestinal ROS generation in *C. elegans* [44] and increased the mRNA expression of sod-1, sod-2, sod-3, ctl-1, and ctl-2 [38,44]. Zhang et al. (2020) reported that silica nanoparticles (SiO_2_ NPs) induced an increase in ROS, which suggests that NPs can affect the oxidative stress levels of *C. elegans* [45]. According to previous research, oxidative stress is interconnected with nanoparticle toxicity [46]. Therefore, significant changes to oxidative stress levels can lead to nanoparticle toxicity. Previous studies also observed that upregulation of oxidative stress-related genes is part of the defense mechanism of the nematodes. Excess free radicals may overwhelm the defense mechanism of *C. elegans*, leading to oxidative stress [37,44,47]. Excessive ROS generation can induce lethal oxidative damage to critical proteins and trigger cell death pathways [48].

In the current study, after exposing the nematodes to B4C-NPs, production of progeny significantly decreased (Figure 2C), and the expression of apoptosis-related genes (the genes of ced-3, ced-9, ced-4, and cep-1) notably increased, particularly at 320 mg/L (Figure 4). The DNA damage checkpoint gene hus-1, which is crucial for cell cycle arrest and apoptosis in response to DNA damage, was significantly upregulated following B4C NP exposure (Figure 4E). These findings suggest that DNA damage may play a role in B4C NP-induced apoptosis. Apoptosis is a programmed cell death process that eliminates unwanted cells during development and removes irreparably damaged cells in adults. The expression of ced-3, ced-4, ced-9, ced-13, hus-1, and cep-1 genes plays a role in regulating apoptosis in *C. elegans* [22,23,24,25]. In adult *C. elegans*, more than 300 germ cells typically undergo apoptosis to eliminate excess reproductive cells through the apoptotic execution machinery involving ced-3, ced-4, and ced-9 [49]. SiO_2_ NPs induced a significant increase in the number of apoptotic gonad cells after 24 h of exposure [45]. Moreover, a meta-analysis by Yin et al. (2024) reported that nanomaterials caused upregulation of ced-3, ced-4, and cep-1, which means that nanomaterials can increase the death risk in nematodes, as well as reducing their survival rate and lifespan [50].

In *C. elegans*, the biosynthesis and transport of serotonin are regulated by the gene expressions of cat-1, cat-4, mod-5, and tph-1. The cat-1 gene (which enables dopamine–sodium symporter activity and serotonin–sodium chloride symporter activity) facilitates dopamine and serotonin transport; cat-4 (which enables GTP cyclohydrolase I activity) is involved in dopamine and tetrahydrobiopterin biosynthesis; mod-5 (which enables serotonin–sodium chloride symporter activity) regulates serotonin uptake; and tph-1 (predicted to enable tryptophan 5-monooxygenase activity) contributes to serotonin biosynthesis and influences various biological processes, including lifespan and locomotory behavior [27,28,29,30,31,32]. As shown in Figure 5, based on the current results, these gene expressions were upregulated at concentrations 40, 160, and 320 mg/L, indicating that the abnormal expression of serotonin- and dopamine-related genes may contribute to the behavioral impairments observed in *C. elegans* following B4C NP exposure. According to a previous study, mutations in gene expressions of tph-1, cat-4, and mod-5 led to a deficiency in the thermotaxis memory behavior of nematodes [51]. Both previous studies and our findings suggest that B4C-NPs may impair neurotransmission, leading to abnormalities in neurobehavioral functions and thermotaxis memory behavior in *C. elegans*.

As illustrated in Figure 6, the results of this study showed a significant increased expressions in the genes of mtl-1, mtl-2, and cyp35a2, implying that B4C-NPs had significant effects on detoxification and drug metabolism in *C. elegans*. Genetic expression of mtl-1, mtl-2, and cyp35a2 is responsible for metal detoxification and drug metabolism [34,35]. The cyp35 genes are involved in various biological processes, such as fatty acid synthesis and storage, xenobiotic metabolism, and stress response in *C. elegans* [34]. A previous study reported an increase in cyp35a2 gene expression in nematodes after treatment with cerium (IV) oxide (CeO_2_), which led to a decrease in fertility and survival rates [52]. The primary functions of metallothioneins (MTs) include metal detoxification, homeostasis regulation, and stress adaptation. The genes mtl-1 and mtl-2 play a role in regulating growth and fertility in *C. elegans* [53,54]. These findings are similar to the results of this study, which showed a significant decline in production of progeny (Figure 2C).

Finally, not many published reports have investigated the adverse effects of in vitro exposure to B4C-NPs [7,8]. Given their potential as a delivery system for future applications in boron neutron capture therapy (BNCT), the safety of B_4_C-NPs should first be assessed through toxicity tests in cellular models. Although BNCT is not directly related to our previous study, the key findings and significant results from BNCT-related reports are summarized in the Supplementary Materials. Türkez et al. (2019) [7] indicated that 20% maximal inhibitory concentration (IC_20_) of 202.525 mg/L B4C-NPs in HPAEpiC cells were suggested as a safe engineering nanomaterial (ENM) for use in medical and pharmacological applications. Based on our findings, B4C-NPs at the IC_20_ dosage evaluated by Türkez et al. (2019) [7] caused neurological, reproductive, and developmental toxicity, shortened longevity, and genotoxicity. We recommend reevaluating B4C-NPs as a safe ENM for widespread use in the medical and pharmacological industries.

Additionally, the limitations of the present study include the following. The toxic effects of the present study were tested in *C. elegans*. Rodent-based models for B4C-NPs toxicity are needed to further assess adverse health effects after humans are exposed to B4C-NPs. Human exposure to B4C-NPs might potentially come primarily from airborne pathways, particularly for workers in certain workplaces or soldiers who might be inadvertently exposed during battle. The B4C-NPs used in this study were a gift from a military research institute in Taiwan, produced for the engineering and manufacture of ENM for armor. It was difficult to obtain complete information about composition and manufacturing process of these B4C-NPs. Furthermore, interactions between NPs and *E. coli* OP50 can confound *C. elegans* toxicity results; Starnes et al. (2016) [40] demonstrated that positively charged NPs agglomerate with *E. coli*, affecting growth and reproduction. Although B4C-NPs may differ in surface charge and no aggregation with *E. coli* was observed in our study, probably because UV-irradiated *E. coli* was coated on plates rather than suspended in M9 medium, future studies should investigate B4C-NPs’ interaction with *E. coli* and include controls to distinguish direct and food-related effects.

## 5. Conclusions

This is the first study to address the nanotoxicity of B4C-NPs using a *C. elegans* model. In this study, prolonged exposure of nematodes to B4C-NPs, particularly at 160 and 320 mg/L, significantly generated toxic effects on locomotion, reproduction, and development, shortened longevity, and caused genotoxicity, including oxidative stress, cell cycle arrest, and apoptosis, affecting neurotransmitter synthesis, transportation, and the biosynthesis of serotonin. 

B4C-NPs are widely used in the military, especially in armor engineering. During the Russia–Ukraine war, soldiers might be inadvertently exposed to severe levels of B4C-NPs, causing adverse health effects in addition to potential injuries during battle. B4C-NPs might be a candidate as a neurotoxic substance to delay neurobehavioral development in *C. elegans* at 40 mg/L, according to the present study. Before medical application, it is necessary to reevaluate their safe use, based on the nanotoxicicity and nanogenotoxicity of B4C-NPs revealed in the present study. According to our findings, the design of future epidemiological or rodent-based animal studies is encouraged.

## Figures and Tables

**Table 1:** Primer sequences.

Gene Code	Forward Primer (5′ to 3′)	Reverse Primer (5′ to 3′)
*C. elegans*		
skn-1	GGACGTCAACAGCAGACTCA	GAGAGCACGTTGATGACGAA
sod-1	TCAGGTCTCCAACGCGATTT	ACCGGGAGTAAGTCCCTTGA
sod-2	GAGGCGGTCTCCAAAGGAAA	CGCTCTTAATTGCGGTGAGC
sod-3	CTCCAAGCACACTCTCCCAG	TCCCTTTCGAAACAGCCTCG
sod-4	GACGCGGTACTTCAGACCAA	CTGGAGGAAGGGATGCTGTC
sod-5	CCGATAAGGTGGTCAGCCTC	CAAAGACTCCTCGGCCTTGT
ctl-1	GTGTCGTTCATGCCAAGGGAG	TGGATTGCGTCACGAATGAAG
ctl-2	TCCCAGATGGGTACCGTCAT	GGTCCGAAGAGGCAAGTTGA
ctl-3	ATGCCAATGCTTCCCCACAT	GCAGGTGGGGTTCCTGATTT
cat-1	CGGTAGAAACTGAAGAACCTG	AGGCATAGTGTAGCCGATTC
cat-4	TCGGAGAAGACATCAATCG	GCTCACAAAGGGAGAACATT
mod-5	ATTATTCAAGCCTATGTTCCAA	GAGATGAGATTCCGACAGT
tph-1	CTGCCGATTCTCCAGTAAAA	ACTACCCTCAACGGCATGTT
cyp35a2	TCGATTTGTGGATGACTGG	AATGGATGCATGACGTTGAA
mtl--1	AGTGCGGAGACAAATGTGAATGC	AGCAGTTCCCTGGTGTTGATGG
mtl-2	TTGTTCCTGCAACACCGGAA	GTTGGCACACTTGCATCCTC
ced-3	GGAGCTTGCTAGAGAGGAACA	TCAGCAAGTCCTTCGTGTCC
ced-4	GCTGATGCTAAAAAGCGAAGA	CGTTGCTGGATTTCCACTGC
ced-9	AGTGATGCTCAGGACTTGCC	AGCCTTGGCTCTTCCCAATC
ced-13	ACACTTTCTCCCGCTGTTGT	TCAGAGTCAAACTCGTCGCA
hus-1	CGGCGGACACTGTTATCTGA	AATTGACGGCCTGGAGTACG
cep-1	AGAATACCCGATTCGCAGGAC	TCGCCATTGCCCAGTATTCC
actin	AGAAGAGCACCCAGTCCTCC	GAAGCGTAGAGGGAGAGGAC

## Data Availability

The original contributions presented in this study are included in the article.

## Supplementary Materials

The following supporting information can be downloaded at https://www.mdpi.com/article/10.3390/toxics13060492/s1, S1. Nanoparticle characterization methods of B4C-NPs [16]; S2. Supporting information reprinted from other studies [3,55,56,57,58,59,60,61,62].
