# Supplementary material for: The Impact of Boron Carbide Nanoparticle (B4C-NPs) Toxicity on Caenorhabditis elegans Models
Source: Toxics. 2025 Jun 12;13(6):492. doi: 10.3390/toxics13060492 (PMC12197407; doi:10.3390/toxics13060492)
Supplement: Supplementary file 1 [file toxics-13-00492-s001.zip › toxics-3604797-supplementary.pdf]

Supplementary Materials

toxics

Manuscript title: The impact of Boron Carbide Nanoparticle (B4C-NP) Toxicity on *Caenorhabditis elegans* Models

Manuscript number: toxics-3604797

Content

S1. Nanoparticle characterization methods of B4C-NPs

S2. The supporting information and reprinted from the other studies

S1. Nanoparticle characterization methods of B4C-NPs

The part of the present study was referenced from the Manimaran's report [16]. Briefly, the B4C-NPs was synthesized using a high-temperature tube furnace. Subsequently, B4C-NPs were characterized by transmission electron microscopy (TEM), X-ray diffraction (XRD), and X-ray photoelectron spectroscopy (XPS). Structural analysis of B4C-NPs was conducted using XRD to obtain crystallographic patterns (confirmed rhombohedral structure of B4C-NPs), XPS to evaluate functionalization states (verified B–B and B–C bonds in B_4C with B 1s peak at ~188.3 eV), and TEM to examine morphological features (showed morphological features and nanoscale distribution).

Reference

16. Manimaran, P.; Chen, S.-M.; Aldossari, S.A.; Rajaji, U.; Hung, K.-Y. Elevated temperature fabrication of copper sulfide nanosphere implemented boron carbide nanocomposite for hyper-sensitive detection of sulfadiazine in water and urine samples. *J. Colloid Interface Sci.* **2025**, *692*, 137503.

S2. The supporting information and reprinted from the other studies

1. Mortensen MW, Sørensen PG, Björkdahl O, Jensen MR, Gundersen HJ, Bjørnholm T. Preparation and characterization of Boron carbide nanoparticles for use as a novel agent in T cell-guided boron neutron capture therapy Appl Radiat Isot. 2006 Mar;64(3):315-24.

   <Summary>

   B4C-NPs were created for use in T cell-guided boron neutron capture therapy (BNCT), a type of cancer treatment. These tiny particles, made by grinding commercial B4C-NPs in different environments, were all smaller than 100 nanometers and showed major changes in their surface and structure. Scientists confirmed these changes using various tests like electron microscopy and spectroscopy. When tested in the lab, melanoma cancer cells that had absorbed these nanoparticles were completely destroyed after being exposed to thermal neutrons. Importantly, the nanoparticles alone didn’t harm the cells, showing they could be a safe and powerful tool for BNCT [55].

2. Mortensen MW, Björkdahl O, Sørensen PG, Hansen T, Jensen MR, Gundersen HJ, Bjørnholm T. Functionalization and cellular uptake of boron carbide nanoparticles. The first step toward T cell-guided boron neutron capture therapy Bioconjug Chem. 2006 Mar-Apr;17(2):284-90.

   <Summary>

   The coated B4C-NPs were successfully taken up by mouse cancer cells—specifically, EL4 thymoma and B16 melanoma cells—at fairly high levels. When these loaded cells were exposed to neutrons, they not only died themselves but also caused nearby untreated cancer cells to stop growing. This shows a "bystander effect," meaning the treated cells can help damage other tumor cells around them. These results are an encouraging first step toward using T cells to deliver boron for a new kind of cancer therapy called BNCT [56].

3. DeLong RK, Mitchell JA, Morris RT, Comer J, Hurst MN, Ghosh K, Wanekaya A, Mudge M, Schaeffer A, Washington LL, Risor-Marhanka A, Thomas S, Marroquin S, Lekey A, Smith JJ, Garrad R, Aryal S, Abdelhakiem M, Glaspell GP. Enzyme and Cancer Cell Selectivity of Nanoparticles: Inhibition of 3D Metastatic

Phenotype and Experimental Melanoma by Zinc Oxide J Biomed Nanotechnol. 2017 Feb;13(2):221-31.

<Summary>

This study looked at how different metal and metal oxide nanoparticles affect two important enzymes—luciferase (Luc) and β-galactosidase (β-Gal)—and how they might be used to treat melanoma. NiO nanoparticles greatly boosted β-Gal activity (over 4 times more than normal). B4C-NPs blocked Luc activity (less than 10% of normal). ZnO and Co_3O_4 nanoparticles slightly increased β-Gal activity. MgO nanoparticles mildly reduced the activity of both enzymes. In cancer cell tests, the nanoparticles killed melanoma cells in this order: ZnO > B_4C ≈ Cu > MgO > Co_3O_4 > Fe_2O_3 > NiO. ZnO NPs also blocked important cancer-related signals, stopped 3D tumor growth, worked as glowing cell labels, and showed good results in mice with melanoma. Overall, this study shows that different nanoparticles can have very specific effects on enzymes and cancer cells, making them useful tools for cancer treatment research and tumor modeling [57].

4. Thomas SE, Comer J, Kim MJ, Marroquin S, Murthy V, Ramani M, Hopke TG, McCall J, Choi SO, DeLong RK. Comparative functional dynamics studies on the enzyme nano-bio interface Int J Nanomedicine. 2018 Aug 8;13:4523-4536.

<Summary>

When tested with a technique called 2-D PLDS, B4C-NPs completely shut down bioluminescence, while Cu and iron oxide (Fe_2O_3) NPs showed unusual, two-phase enzyme activity changes—Cu caused a big shift of 271%. Computer simulations showed: (1) Metal oxide nanoparticles (like Cu and Fe_2O_3) form metal hydroxides on their surfaces. (2) B4C-NPs form boronic or borinic acid groups. These surface properties were confirmed with tests like zeta potential and thin-layer chromatography, which revealed that nanoparticle stability depends on how well they interact—ionic > polar > non-polar. In short, B4C-NPs and Cu nanoparticles have unique effects on enzymes, suggesting they could influence important biological processes and deserve more study in health and disease research [58].

5. Kozień D, Szermer-Olearnik B, Rapak A, Szczygieł A, Anger-Góra N, Boratyński

J, Pajtasz-Piasecka E, Bućko MM, Pędzich Z. Boron-Rich Boron Carbide Nanoparticles as a Carrier in Boron Neutron Capture Therapy: Their Influence on Tumor and Immune Phagocytic Cells. Materials (Basel). 2021 Jun 2;14(11):3010.

<Summary>

B4C-NPs (averaging 80 nm)were coated with human antibodies (IgG), some of which were labeled with a fluorescent tag, to see how they interact with cells. Researchers tested these coated nanoparticles on two types of mouse cells: (1) MC38 colon cancer cells; (2) RAW 264.7 immune cells (macrophages). The immune cells (macrophages) took in the B4C-IgG-NPs, showing active uptake. The cancer cells showed very little interaction with the nanoparticles. These B4C-NPs are better at targeting immune cells than cancer cells, which could be useful for immune-based therapies [59].

6. Wang Y, Reina G, Kang HG, Chen X, Zou Y, Ishikawa Y, Suzuki M, Komatsu N. Polyglycerol Functionalized ^{10}B Enriched Boron Carbide Nanoparticle as an Effective Bimodal Anticancer Nanosensitizer for Boron Neutron Capture and Photothermal Therapies Small. 2022 Sep;18(37):e2204044.

<Summary>

BNCT is a non-invasive way to treat cancer by using a special form of boron (^{10}B) that breaks apart when hit by neutrons, killing nearby cancer cells. Two boron-based drugs are already used, but they don't stay in tumors well and don't target them precisely. To improve this, researchers have created boron-containing nanoparticles, especially boron carbide (^{10}B4C), which holds a lot of boron. However, using it in living systems has been difficult. Interestingly, boron carbide can also absorb light and turn it into heat, which could help kill tumors when combined with BNCT.

In this study, ^{10}B4C-NPs were coated with polyglycerol (PG) to help them spread well in the body. These new particles (^{10}B4C-PG) met three important BNCT requirements: (1) Safe for healthy tissue (low toxicity); (2) Delivered enough boron to tumors (over 20 ppm); (3) Had at least 3 times more boron in tumors than in blood. When tested in animals, combining neutron therapy with near-infrared light stopped tumor growth—and even wiped some tumors out—showing that ^{10}B4C-PG could be a powerful new cancer treatment using both BNCT and heat therapy [60].

7. Kozień D, Żeliszewska P, Szermer-Olearnik B, Adamczyk Z, Wróblewska A, Szczygieł A, Węgierek-Ciura K, Mierzejewska J, Pajtasz-Piasecka E, Tokarski T, Cios G, Cudziło S, Pędzich Z. Synthesis and Characterization of Boron Carbide Nanoparticles as Potential Boron-Rich Therapeutic Carriers Materials (Basel). 2023 Oct 2;16(19):6534.

<Summary>

B4C-NPs were made by heating a mix of boron and carbon powders to 1650 °C for 2 hours in argon gas. Scientists then studied their shape and structure using tools like SEM, TEM, AFM, XRD, and DLS. They also measured important features, including how the particles move in liquid, their size in solution, surface charge, and electrical behavior. When tested on human and mouse cells, the smaller particles were more toxic, showing that size matters for safety. The goal of the study was to see if these B_4C nanoparticles could be useful for BNCT—a promising cancer treatment [3].

8. Wróblewska A, Szermer-Olearnik B, Szczygieł A, Węgierek-Ciura K, Mierzejewska J, Kozień D, Żeliszewska P, Kruszakin R, Migdał P, Pędzich Z, Pajtasz-Piasecka E. Macrophages as carriers of boron carbide nanoparticles dedicated to boron neutron capture therapy J Nanobiotechnology. 2024 Apr 15;22(1):183.

<Summary>

Macrophages, from both lab-grown cells and bone marrow, interacted more strongly with B4C-NPs than dendritic cells. The larger B4C-NPs caused more cell damage and triggered more inflammation. In contrast, the smaller B4C-NPs were less toxic, allowed macrophages to move more easily, and still activated them effectively. These findings suggest that B4C-NPs are a safer and better option for using macrophages to deliver boron in BNCT- helping target tumors more precisely [62].

9. Xu S, Yu Y, Zhang B, Zhu K, Cheng Y, Zhang T. Boron carbide nanoparticles for boron neutron capture therapy RSC Adv. 2025 Apr 7;15(14):10717-10730.

<Summary>

Researchers compared modified B4C-NPs with unmodified ones to see how they behaved in the body. The modified version (called B_4C-APTES-PEG2K-FA) had better properties—it was safer for blood cells, less toxic to normal cells, and easier for cells to absorb. When tested in mice, the B4C-NPs mostly gathered in the lungs, followed by the liver, spleen, kidneys, heart, and tumors. Very little ended up in the brain and muscles. After 24 hours, the tumors had high levels of boron (50 μg/g dry tissue)—with tumor-to-muscle and tumor-to-brain ratios over 3. This shows that the modified B4C-NPs are promising for BNCT because they safely deliver a lot of boron to tumors and can be further customized for better targeting [63].

References

55. M.W. Mortensen, P.G. Sørensen, O. Björkdahl, M.R. Jensen, H.J. Gundersen, T. Bjørnholm, Preparation and characterization of Boron carbide nanoparticles for use as a novel agent in T cell-guided boron neutron capture therapy, *Appl Radiat Isot*. **2006**, 64, 315-24.
56. M.W. Mortensen, O. Björkdahl, P.G. Sørensen, T. Hansen, M.R. Jensen, H.J. Gundersen, T. Bjørnholm, Functionalization and cellular uptake of boron carbide nanoparticles. The first step toward T cell-guided boron neutron capture therapy, *Bioconjug Chem*. **2006**, 17, 284-90.
57. R.K. DeLong, J.A. Mitchell, R.T. Morris, J. Comer, M.N. Hurst, K. Ghosh, A. Wanekaya, M. Mudge, A. Schaeffer, L.L. Washington, A. Risor-Marhanka, S. Thomas, S. Marroquin, A. Lekey, J.J. Smith, R. Garrad, S. Aryal, M. Abdelhakiem, G.P. Glaspell, Enzyme and Cancer Cell Selectivity of Nanoparticles: Inhibition of 3D Metastatic Phenotype and Experimental Melanoma by Zinc Oxide, *J Biomed Nanotechnol*. **2017**, 13, 221-31.
58. S.E. Thomas, J. Comer, M.J. Kim, S. Marroquin, V. Murthy, M. Ramani, T.G. Hopke, J. McCall, S.O. Choi, R.K. DeLong, Comparative functional dynamics studies on the enzyme nano-bio interface, *Int J Nanomedicine*. **2018**, 13, 4523-4536.
59. D. Kozień, B. Szermer-Olearnik, A. Rapak, A. Szczygieł, N. Anger-Góra, J. Boratyński, E. Pajtasz-Piasecka, M.M. Bućko, Z. Pędzich, Boron-Rich Boron Carbide Nanoparticles as a Carrier in Boron Neutron Capture Therapy: Their Influence on Tumor and Immune Phagocytic Cells, *Materials (Basel)*. **2021**, 14, 3010.
60. Y. Wang, G. Reina, H.G. Kang, X. Chen, Y. Zou, Y. Ishikawa, M. Suzuki, N. Komatsu, Polyglycerol Functionalized (10) B Enriched Boron Carbide Nanoparticle as an Effective Bimodal Anticancer Nanosensitizer for Boron Neutron Capture and Photothermal Therapies, *Small*. **2022**, 18, e2204044.
61. A. Wróblewska, B. Szermer-Olearnik, A. Szczygieł, K. Węgierek-Ciura, J. Mierzejewska, D. Kozień, P. Żeliszewska, R. Kruszakin, P. Migdał, Z. Pędzich, E. Pajtasz-Piasecka, Macrophages as carriers of boron carbide nanoparticles dedicated to boron neutron capture therapy, *J Nanobiotechnology*. **2024**, 22, 183.
62. S. Xu, Y. Yu, B. Zhang, K. Zhu, Y. Cheng, T. Zhang, Boron carbide nanoparticles for boron neutron capture therapy, *RSC Adv*. **2025**, 15, 10717-10730.
